# Black spot diseases in seven commercial fish species from the English Channel and the North Sea: infestation levels, identification and population genetics of *Cryptocotyle* spp.[Fn FN1]

**DOI:** 10.1051/parasite/2023028

**Published:** 2023-07-06

**Authors:** Maureen Duflot, Pierre Cresson, Maéva Julien, Léa Chartier, Odile Bourgau, Marialetizia Palomba, Simonetta Mattiucci, Graziella Midelet, Mélanie Gay

**Affiliations:** 1 ANSES, Laboratory for Food Safety 62200 Boulogne-sur-Mer France; 2 University of Littoral Côte d’Opale Boulogne-sur-Mer France; 3 Ifremer, RBE/HMMN, Laboratoire Ressources Halieutiques Manche Mer du Nord 62200 Boulogne-sur-Mer France; 4 Department of Ecological and Biological Sciences, Tuscia University, Viale dell’Università s/n 01100 Viterbo Italy; 5 Department of Public Health And Infectious Diseases, Section of Parasitology, Sapienza University of Rome P.le Aldo Moro, 5 00185 Rome Italy

**Keywords:** *Cryptocotyle*, Trematode, Commercial fish species, Epidemiological study, Parasitological descriptors

## Abstract

Fish are often speckled with “black spots” caused by metacercarial trematode infection, inducing a host response. *Cryptocotyle* spp. (Opisthorchiidae) are among the parasites responsible for this phenomenon. So far, the impact on human health is still unknown. In addition, few publications dealing with black spot recovery, identification, distribution and diversity among commercially important fish are available. Moreover, “black spots” have been observed by fishermen on marine fish, revealing an appreciable but unquantified presence in consumed fish. An epidemiological survey of 1,586 fish from seven commercial species (herring, sprat, whiting, pout, dab, flounder, and plaice) was conducted in the Eastern English Channel and the North Sea in January 2019 and 2020. Encysted metacercariae were found in 325 out of 1,586 fish, with a total prevalence of 20.5%. Intensity of infection varied from 1 to 1,104 parasites. The recorded encysted metacercariae were identified either by microscopic examination or with molecular tools. Partial sequences of the mtDNA *cox1* gene and of the rDNA *ITS* region were obtained. Two species of *Cryptocotyle*, *Cryptocotyle lingua* (Creplin, 1825) and *Cryptocotyle concava* (Creplin, 1825) were found. Metacercariae belonging to other trematode families were also identified. Molecular phylogenetic analysis and haplotype network construction were performed to confirm the identification and to study the potential presence of different populations of *Cryptocotyle* spp. This survey enabled us to describe the distribution of two species of *Cryptocotyle* in the English Channel and North Sea ecosystems. The observed differences in infestation levels between fish species and geographical areas will contribute to better understanding of the ecology of these parasites.

## Abstract (fr)

Les poissons sont souvent parsemés de « points noirs » causés par une infection par des métacercaires de trématodes induisant une réponse de l’hôte. Les *Cryptocotyle* spp. (Opisthorchiidae) font partie des parasites responsables de ce phénomène. Jusqu’à présent, leur impact sur la santé humaine est inconnu. De plus, il existe peu de publications traitant de la récupération, l’identification, la distribution et la diversité des « points noirs » parmi les poisons d’importance commerciale. Par ailleurs, des observations de « points noirs » sur les poissons marins ont été constatées par les pêcheurs révélant une présence assez importante mais non quantifiée dans les poissons consommés. Une enquête épidémiologique portant sur 1 586 poissons de sept espèces commerciales (hareng, sprat, merlan, tacaud, limande, flet et plie) a été menée en Manche orientale et en Mer du Nord, en janvier 2019 et 2020. Des métacercaires enkystées ont été trouvées chez 325 poissons parmi 1 586, avec une prévalence totale de 20,5 %. L’intensité de l’infection variait de 1 à 1 104 parasites. Les métacercaires enkystées répertoriées ont été identifiées soit par examen microscopique, soit avec des outils moléculaires. Des séquences partielles du gène *cox1* de l’ADNmt et de la région *ITS* de l’ADNr ont été obtenues. Deux espèces de *Cryptocotyle*, *Cryptocotyle lingua* (Creplin, 1825) et *Cryptocotyle concava* (Creplin, 1825) ont été trouvées. Des métacercaires appartenant à d’autres familles de trématodes ont également été identifiées. Une analyse phylogénétique moléculaire et la construction d’un réseau d’haplotypes ont été effectuées pour confirmer l’identification et étudier la présence potentielle de différentes populations de *Cryptocotyle* spp. Cette étude a permis de décrire la distribution de deux espèces de *Cryptocotyle* dans les écosystèmes de la Manche et de la Mer du Nord. Les différences observées dans les niveaux d’infestation entre les espèces de poissons et les zones géographiques contribueront à une meilleure compréhension de l’écologie de ces parasites.

## Introduction

Fish harbor many pathogens – including parasites – that may affect cultured or wild fish. Their presence may impact fish production and, for some of them, they may be a threat to human health. More than 50 to 60 million people are annually reported to be infected by foodborne trematode infections around the world [15]. Humans can be infected by ingestion of raw, undercooked or pickled fish containing metacercariae. Infection by members of the superfamily Opisthorchioidea causes a wide range of impacts on human health, from very severe human disease, such as in infection by *Clonorchis sinensis* or *Opisthorchis viverrini*, to unknown zoonotic potential for other species [14, 15, 22]. These parasites have a complex life cycle. Commonly they have a mollusc as first intermediate host, in which several larval stages sequentially develop (miracidium, sporocyst, redia, and cercaria). A fish acts as second intermediate host, in which the cercariae evolve into metacercaria. Vertebrate animals, including mammals and birds, complete the life cycle [28, 72]. Many trematode species can encyst at the metacercariae stage in marine and freshwater fish, and some of them cause black spot disease [1, 5, 24, 44]. Additionally, some digeneans, such as *Apophallus* Lühe, 1906 [73], *Cryptocotyle* Lühe, 1899 [17, 78], *Haplorchis* Looss, 1899 [61] and *Stellantchasmus* Onji & Nishio, 1916 [16] infect marine fish, their intermediate host. Their swimming cercariae encyst, develop into the metacercarial stage, and cause immune cutaneous black spot. Black spot disease is an immune response towards encysted metacercariae, due to the concentration of melanomacrophages at the infection site [21, 78]. These black spots may induce esthetic problems that lower the commercial value of the fish [44]. Some Opisthorchioidea parasites are known to have zoonotic potential [15, 56, 74]. As *Cryptocotyle* spp. belong to the Opisthorchioidea superfamily, they have a potential zoonotic nature, but their impact on human health is poorly known [13, 15]. In addition, few data are available regarding their distribution among commercially important fish species. Therefore, one of the first steps in risk assessment is the acquisition of knowledge and fundamental data on the distribution of this parasite in different fish species and different geographical areas, before any zoonotic potential assessment.

Reports on parasite fauna in fish from the English Channel and North Sea are numerous but, to date, few have dealt with encysted metacercariae diseases [32, 60]. To study the circulation of *Cryptocotyle* spp. and other black spot-causing parasites in fish, the infestation levels of black spot in seven fish species in five geographical areas, covering the eastern English Channel and the south of the North Sea (*i.e.*, south of < 55 °N), were determined by fish dissection to isolate and identify metacercariae. The seven fish species were selected based on their former description as hosts for *Cryptocotyle* spp., and their biology, behavior, and position in these ecosystems. The first two species (*Clupea harengus* and *Sprattus sprattus*) are medium-small sized species that play an important role in the food web. These zooplankton feeding species are considered pivotal between primary production and top predators, and thus play a major role in ecosystem functioning [54]. Herring (*C. harengus*) has also been a major commercial fish species in the area for centuries, while sprat (*S. sprattus*) abundance and catches have increased in recent years, potentially because of its larger ecological niche [19, 36]. Two species of gadoids (*Merlangius merlangus* and *Trisopterus luscus*) and three species of flatfish (*Limanda limanda*, *Platichthys flesus*, and *Pleuronectes platessa*) were chosen as they are relevant in fisheries. As an example, annual landings in the North Sea and English Channel have been between 50 kt and 80 kt for plaice (*P. platessa*), between 3 kt and 7 kt for dab (*L. limanda*), and around 15 kt for whiting (*M. merlangius)* since 2010 [35, 37, 38]. In addition, they occupy different positions in the water column: herring and sprat are strictly pelagic, gadoids are bentho-demersal (*i.e.*, able to move vertically in the column), and flatfish species are considered to be purely benthic.

The selected species have already been described as *Cryptocotyle* spp. hosts [7, 32, 59, 62, 65, 67, 91]. The present study aimed at assessing the prevalence, intensity, and abundance of encysted metacercariae in the seven selected fish species collected in the Eastern English Channel and North Sea. Moreover, preferential anatomical location of black spot disease was characterized. Metacercariae were identified molecularly or morphologically. Molecular examinations were completed with phylogenetic analyses incorporating species of the superfamily Opisthorchioidea Loss, 1899 and an analysis of genetic diversity within the species *C. lingua*. This study provides the first evidence of *Cryptocotyle* spp. distribution in the Eastern English Channel and North Sea.

## Material and methods

### Fish samples

**Figure 1:**
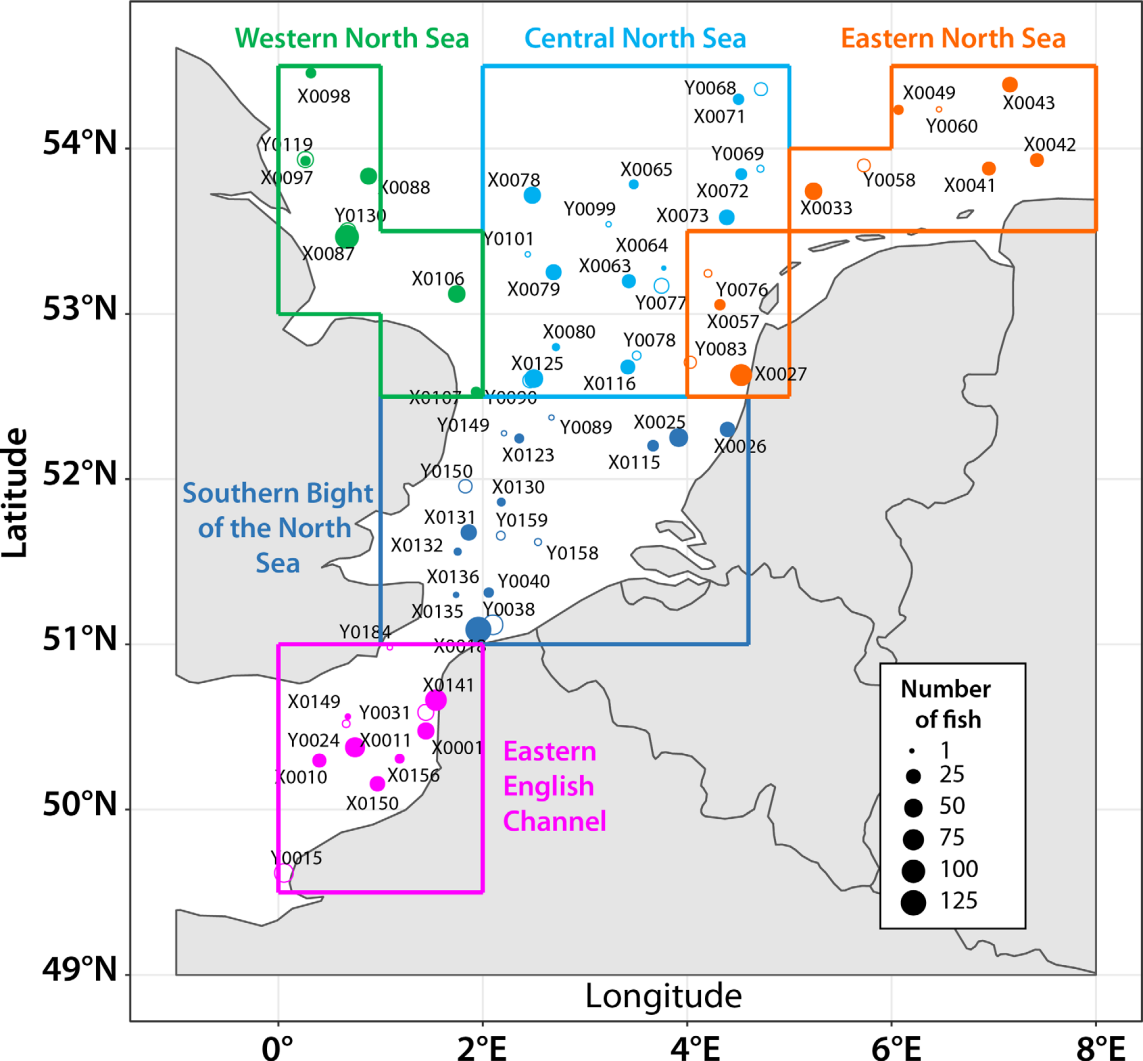
Sampling locations in the English Channel and North Sea with indications of number of sampled fish at each station. Size of the circle is proportional to the total number of fish (*n* fish) of the five species sampled at each station. Stations names with a code beginning with an X and with full circle were sampled in 2019 and by a Y and empty circle in 2020.

Seven fish species were selected for this study: herring *Clupea harengus* Linnaeus, 1758, sprat *Sprattus sprattus* (Linnaeus), whiting *Merlangius merlangus* Linnaeus, pout *Trisopterus luscus* (Linnaeus), dab *Limanda limanda* (Linnaeus), flounder *Platichthys flesus* (Linnaeus), and plaice *Pleuronectes platessa* Linnaeus. All species were sampled in 2019 while whiting and pout only were collected in 2020. A total of 1,163 and 423 specimens were collected by bottom trawling during the International Bottom Trawl Survey in the English Channel and North Sea in January 2019 and 2020, respectively [39, 40]. The initial protocol included the collection of 40 individuals in each geographical area. The sampling area was divided into five sub-areas defined based on environmental characteristics (temperature, salinity) (Fig. 1). Individuals of all species were sampled in all areas, except for pout in the east North Sea in both years (Table 1). For 2019 sampling, all fish were frozen at −20 °C rapidly on board. For 2020 sampling, individuals were eviscerated and kept fresh at 1 °C for a maximum of 11 days until parasite inspection at the laboratory.

**Table 1:** Geographical origin and biometric data of fish species, including weight and length ± SD (min–max).

Species	Fishing year	Number of fish^a^					Weight (g)	Total length (mm)	Parasite identification methods
		EEC	SBNS	ENS	CNS	WNS			
Herring	2019	21	40	43	40	39	41.72 ± 36.37 (2–183)	170.93 ± 53.98 (35–306)	Molecular
Sprat	2019	40	42	38	44	43	6.89 ± 3.00 (1–19)	99.58 ± 13.57 (54–135)	Molecular
Whiting	2019	40	40	30	40	40	218.70 ± 136.43 (16–674)	258.26 ± 64.83 (126–405)	Molecular
	2020	80	39	41	66	40	143.00 ± 109.29 (4–882)	249.68 ± 61.99 (98–450)	Morphological & Molecular
Pout	2019	39	40	0	13	23	146.18 ± 109.83 (16–773)	219.64 ± 45.04 (126–367)	Molecular
	2020	26	68	0	24	39	102.68 ± 61.57 (28–421)	204.05 ± 35.79 (140–317)	Morphological & Molecular
Dab	2019	40	40	40	40	40	91.64 ± 51.82 (8–249)	2020.23 ± 35.78 (100–296)	Molecular
Flounder	2019	26	38	36	15	2	207.94 ± 108.65 (18–652)	263.48 ± 47.03 (125–420)	Molecular
Plaice	2019	35	34	25	34	23	163.75 ± 188.80 (3–1314)	229.86 ± 98.13 (77–524)	Molecular

a Number of fish per area; EEC: eastern English Channel, SBNS: Southern Bight of the North Sea, ENS: eastern North Sea, CNS: central North Sea, WNS: western North Sea.

### Evaluation of infection and parasitological descriptors

**Figure 2:**
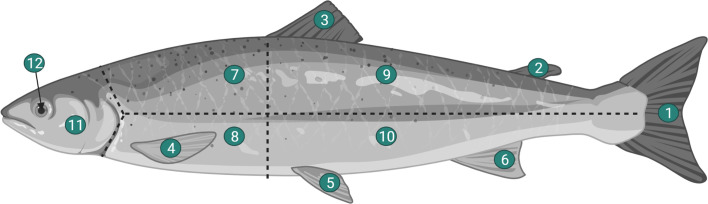
Definition of the different areas of a fish: (1) caudal fin, (2) 2nd dorsal fin, (3) 1st dorsal fin, (4) pectoral fin, (5) pelvic fin, (6) anal fin, (7) dorsofrontal area, (8) ventrofrontal area, (9) dorsoposterior area, (10) ventroposterior area, (11) opercula, and (12) eye; according to Buchmann [10].

Parasitological infection was first assessed by macroscopic examination of fish. The presence of cutaneous black spots was recorded for each defined body area on both sides of each fish (Fig. 2), according to the method used by Duflot *et al.* [21]. Fish that exhibited one or more of the typical black spots formed around encysted metacercariae were recorded as being “infected”, and individuals with no visible spots were recorded as “uninfected”. Three parasitological descriptors were used in the present study, following definitions from Bush *et al.* [12]. In particular, prevalence is the number of fish infected divided by the total number of host fish examined, abundance is the number of black spots divided by the number of fish examined, and intensity is the number of black spots on an infected fish. The data were analyzed with R software 4.0.2 (R Core Team, 2020) and the ggplot2 package [86].

### Isolation of metacercariae

For frozen fish (2019 sampling), the samples were thawed slowly at 1 °C overnight. For each infected fish, a maximum of 15 metacercariae were isolated through dissection and/or partial digestion of skin and subcutaneous muscle (thickness ~ 5 mm). Partial digestion was achieved in a Petri dish by adding a pepsin/HCl/saline solution in excess until tissues were fully immersed [7]. The Petri dish was placed on a hot plate at 37 °C (± 1) for 5–10 min, depending on the fish species. Metacercariae were visually isolated from the tissues under a stereomicroscope (SZX16, Olympus Corporation, Tokyo, Japan), with micro-pliers and a scalpel. Each metacercaria was placed in a separate well on a 96-well plate.

For each fresh individual (2020 sampling), the most infected area of the skin and of the underskin muscle of each infected fish was selected for the isolation of metacercariae. The optimized artificial digestion method described by Duflot *et al.* [21] (D4 method) was used to isolate the metacercariae from the fish tissues. Briefly, skin and subcutaneous muscle were digested separately in a pepsin solution at 37 °C for 1 h on an orbital agitator. Then encystment of each metacercaria was carried out by trypsin treatment at room temperature for 1 h.

### Identification of metacercariae

All the metacercariae isolated from thawed fish were identified by the molecular method. For the parasites isolated from fresh fish, one third of the metacercariae were identified based on morphological criteria (with a maximum of 5 metacercariae per fish), and the other two thirds were identified using the molecular method (with a maximum of 15 per fish). Observation of parasites with the same general morphology under an optic microscope (Ts2 Nikon Eclipse, Nikon, Tokyo, Japan) enabled us to divide samples between the two types of identification methods, morphological and molecular.

Morphological identification was performed according to the protocol described by Duflot *et al.* [20]. Identifications of metacercariae were based on general microscopic observation and measurements of classical characteristics of Opisthorchioidea trematodes on each excysted metacercaria mounted on a slide after staining with hematoxylin. Supplemental measurements were acquired such as the oral and ventrogenital sucker width, the distance between the oral sucker and the pharynx, and the pharynx length and width. Parasites were characterized (×100–200 magnification) under a Leica DLMB microscope with a Leica DC300 camera (Leica, Wetzlar, Germany). Identifications were based on the descriptions of Borges *et al.* [7], Casalins *et al.*[13], Gibson [27], Goncharov *et al.* [30], Linton [53], Ransom [66], Stunkard [80], and Tatonova and Besprozvannykh [83].

Molecular identification was carried out according to a protocol of DNA extraction, PCR amplification, and Sanger sequencing on a partial region of the mtDNA *cox1* gene (≈350 bp) and the rDNA *ITS1-5.8S-ITS2* region (≈1,200 bp) described by Duflot *et al.* [20]. Target regions were amplified using the primer pairs JB3/JB4.5 [9] and BD1/28S1R [77], respectively. PCR products of the expected size were sequenced twice and from both sides (forward and reverse), using Sanger sequencing (Genoscreen, Lille, France) with the same primers. Each obtained consensus sequence was subjected to a BLAST search [3], after visualization in BioEdit 7.0.9.0 software [33], clarification of ambiguous bases, and Clustal W alignment using MEGA 10.1.8 [45]. All sequences the diversity of were submitted to GenBank and assigned accession numbers.

### Phylogenetic analysis and genetic diversity of *Cryptocotyle lingua* populations

The alignments were trimmed to the length of the shortest sequence. Trees were built with reference sequences of digeneans belonging to the superfamily Opisthorchioidea and with *Fasciola* sp. as outgroups (Table 2). Phylogenetic analyses were conducted in MEGA 10.1.8 using the Maximum Likelihood (ML), Neighbor-Joining (NJ) and Minimum Evolution (ME) methods, with 1,000 bootstrap replications. The most suitable fit model for each targeted marker was determined using the corrected Akaike Information Criterion (AICc) and the Bayesian Information Criterion (BIC) on the 24 models tested in MEGA 10.1.8. *Cox1* and *ITS* sequences were fitted to the JC model. Phylogenetic relationships under Bayesian inference (BI) were also generated in MrBayes v3.2.7 [34]. Two independent runs were performed for 10,000,000 generations and sampled every 500th generation. The burn-in was set for the first 25% of the sampled trees. Bayesian analyses were executed online on NGPhylogeny.fr [52].

**Table 2:** Molecular sequences used as references.

Species	Reference	GenBank accession numbers	
		*cox1*	*ITS*
*Clonorchis sinensis*	Lee and Huh [50]	AF181889	–
	Qiu *et al.* [64]	–	MK450527
*Cryptocotyle concava*	Gonchar [29]	MT422290; MT422303	–
*Cryptocotyle lata*	Tatonova and Besprozvannykh [83]	–	MH025622-MH025623
*Cryptocotyle lingua*	Borges *et al.* [7]	KJ711861–KJ711862	KJ641518–KJ641519
	Blakeslee *et al.* [6]	EU876357–EU876411	–
	Duflot *et al.* [20]	MW542531–MW542549	MW544135–MW544136
*Cryptocotyle micromorpha*	Presswell and Bennett [63]	OL504983	–
*Haplorchis taichui*	Lee *et al.* [49]	KF214770	–
	Le *et al.* [47]	–	KX815126
*Opisthorchis sudarikovi*	Suleman *et al.* [81]	–	MK227161
*Opisthorchis viverrini*	Thaenkham *et al.* [84]	HQ328544	–
Outgroup			
*Fasciola hepatica*	Reaghi *et al.* [68]	MT951585–MT951587	–
	Le *et al.* [48]	–	MN970007
*Fasciola gigantica*	Le *et al.* [48]	–	MN970008

Two analyses of molecular variance were carried out on *Cryptocotyle lingua* samples to assess the presence of different populations. The first analysis was based on the *Cryptocotyle* spp. populations from the different geographical areas, the second on the different fish species. Pairwise genetic differentiation of *C. lingua* was estimated with the fixation index (*F*_st_), using ARLEQUIN 3.5 software [25]. This parameter ranges between 0 and 1, in which *F*_st_ = 0 indicates no differentiation between the populations, and *F*_st_ = 1 means complete differentiation among the sequences of the different populations. Pairwise comparisons of *F*_st_ (assuming that *p* < 0.05 indicates a significant difference) were based on 1,000 permutations of the data matrix. Then, Tajima’s D neutrality test [82] and Fu’s Fs [26] were calculated to verify the null hypothesis of selective neutrality, using DnaSP 6.12.03 software [76].

Estimation of the population genetic diversity of *C. lingua* among sampling areas and among fish species was inferred from mtDNA *cox1* gene and *ITS* region rDNA sequence data sets with the following parameters: number of haplotypes (*Nh*), nucleotide diversity (*π*), haplotype diversity (*Hd*), average number of differences (*K*), number of polymorphic sites (*S*). All parameters were estimated using DnaSP 6.12.03 software [71].

Haplotype network constructions were carried out using PopART 1.7 software [51] based on *cox1* sequences gene (269 bp). Network calculation was realized with the TCS model [18].

## Results

### Black spot infection data

#### Prevalence of infection

**Figure 3:**
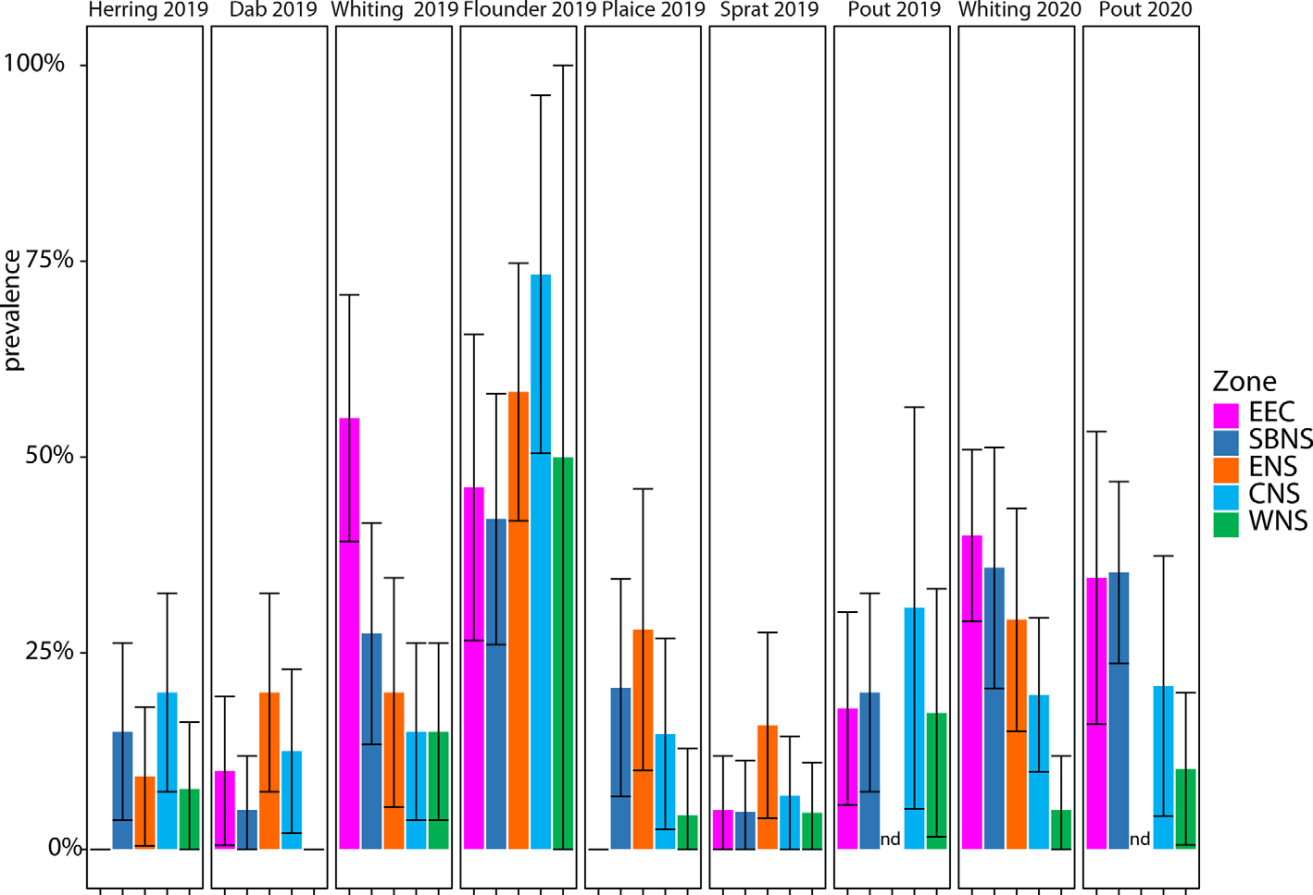
Prevalence of black spot in each fish species by geographical areas. EEC: eastern English Channel, SBNS: Southern Bight of the North Sea, ENS: eastern North Sea, CNS: central North Sea, WNS: western North Sea. Error bar = SD. nd: No sampled individual.

Infection by encysted metacercariae was observed in all sampled areas and for all fish species considered. Of the 1,586 sampled fish, 325 fish were parasitized, an overall prevalence of 20.5%. The prevalence of encysted metacercariae in the different fish species as well as in the different geographical areas was highly variable (Fig. 3). Prevalence values were the highest for *P. flesus* (52.1% in 2019), *M. merlangius* (26.8% and 27.8% in 2019 and 2020, respectively) and *T. luscus* (20.0% and 26.8% in 2019 and 2020, respectively).

Similar prevalence tendencies were observed between the geographical areas in the two sampling years. For whiting samplings, the eastern English Channel was the most infected area, followed by the Southern Bight of the North Sea, the eastern North Sea, central North Sea, and western North Sea. For pout, prevalences were similar in the eastern English Channel and the Southern Bight of the North Sea (about 20% in 2019, and about 35% in 2020). Again, similar trends were observed for prevalences in pout in the central and western North Sea, but with slightly more variation. Pelagic species, *Sprattus sprattus* and *Clupea harengus*, were the least commonly infected fish species overall and within all the geographic areas, with always less than 20% of the fish exhibiting black spots.

#### Abundance and intensity

The abundances or intensities of encysted metacercariae followed the same trends as prevalence values (Table 3). Regardless of the sampling area, whiting and pout were the most infected species, with mean abundances of 8.7 and 9.4 black spots per fish, respectively. Patterns were not consistent between 2019 and 2020 for whiting and pout. For whiting, the Southern Bight of the North Sea and the eastern English Channel were the most infected areas in 2019 (48.7 and 43.2 black spots per fish on average, respectively) while the central and eastern North Sea were the most infected in 2020 (181.2 and 69.6 black spots per fish, respectively). For pout, the eastern English Channel and central North Sea were the most infected geographical areas in 2019 (114.4 and 27.8 spots per fish on an average, respectively), while the central North Sea and the Southern Bight of the North Sea were the most infected in 2020 (55.8 and 42.0 spots per fish). All the targeted fish species sampled in the Southern Bight, eastern North Sea, and central North Sea were parasitized. In the eastern English Channel, herring and plaice were not infected. Likewise, in the western North Sea, dab was not infested. Looking at maximum black spot infection, in 2019, the highest infected individuals of whiting and pout were caught in the eastern English Channel in 2019 (43.2 and 114.4 black spots per fish on average, respectively), and in the central North Sea and the Southern Bight of the North Sea in 2020 (181.2 and 42.0 black spots per fish on average, respectively).

**Table 3:** Mean intensity (I), Abundance (A) and Maximum number of black spots per fish (M) of the different geographic areas, eastern English Channel (EEC), Southern Bight of the North Sea (SBNS), eastern North Sea (ENS), central North Sea (CNS), and western North Sea (WNS) (mean ± SD). (–) No sampled individual.

		EEC			SBNS			ENS			CNS			WNS			All areas		
		I	A	M	I	A	M	I	A	M	I	A	M	I	A	M	I	A	M
2019	Whiting	43.2 ± 57.4	23.8 ± 42.6	555	48.7 ± 81.2	13.4 ± 42.6	454	16.0 ± 22.1	3.2 ± 9.9	68	7.3 ± 8.5	1.1 ± 3.3	28	4.2 ± 2.9	0.6 ± 1.1	10	32.4 ± 30.2	8.7 ± 15.6	555
	Sprat	1.0 ± 0.0	0.1 ± 0.0	1	1.0 ± 0.0	0.0 ± 0.0	1	1.7 ± 0.8	0.3 ± 0.8	3	1.0 ± 0.0	0.1 ± 0.0	1	2.0 ± 2.0	0.1 ± 0.4	3	1.4 ± 0.4	0.1 ± 0.1	3
	Herring	0.0	0.0	0	1.5 ± 0.7	0.2 ± 0.3	3	1.3 ± 0.5	0.1 ± 0.5	2	2.6 ± 1.8	0.5 ± 0.8	8	1.7 ± 1.3	0.1 ± 0.4	3	1.9 ± 0.8	0.2 ± 0.3	8
	Pout	114.4 ± 167.7	20.5 ± 71.0	608	10.3 ± 18.2	2.1 ± 8.1	74	–	–	–	27.8 ± 51.5	8.5 ± 28.6	105	22.0 ± 20.4	3.8 ± 8.5	45	47.0 ± 53.0	9.4 ± 23.7	608
	Dab	3.3 ± 4.5	0.3 ± 1.4	10	12.5 ± 21.0	0.6 ± 4.7	23	2.8 ± 1.4	0.55 ± 1.4	6	3.4 ± 3.4	0.4 ± 1.2	10	0.0	0.0	0	4.1 ± 2.5	0.4 ± 0.8	23
	Flounder	3.4 ± 2.8	1.6 ± 1.9	14	12.0 ± 7.9	5.1 ± 5.1	64	7.0 ± 4.0	4.1 ± 4.0	33	16.6 ± 26.3	12.2 ± 22.5	148	1.0	0.5	1	9.3 ± 5.3	4.8 ± 3.9	148
	Plaice	0.0	0.0	0	2.4 ± 1.9	0.5 ± 0.9	8	1.4 ± 0.6	0.4 ± 0.6	3	1.4 ± 0.5	0.2 ± 0.2	2	3.0	0.1	3	1.9 ± 0.7	0.2 ± 0.3	8
2020	Whiting	27.9 ± 47.6	11.2 ± 30.1	229	25.3 ± 72.0	9.1 ± 43.1	180	69.6 ± 58.1	20.4 ± 58.1	339	181.2 ± 5.8	35.7 ± 2.6	1104	5.5 ± 5.0	0.3 ± 1.1	10	60.9 ± 35.2	16.7 ± 18.4	1104
	Pout	3.0 ± 147.9	1.0 ± 87.0	7	42.0 ± 10.5	14.8 ± 6.2	306	–	–	–	55.8 ± 46.1	11.6 ± 21.0	257	7.8 ± 20.4	0.8 ± 6.5	25	32.0 ± 22.1	8.6 ± 11.4	306

#### Site of infection by *Cryptocotyle* spp.

Some areas of the fish body exhibited more infection by encysted metacercariae than others (Fig. 2 and Supplementary data 1), specifically areas 1 (caudal fin), 7 (dorsofrontal area), 9 (dorsoposterior area), 10 (ventroposterior area) and 11 (opercula) (Supplementary data 1). Areas 2 (second dorsal fin), 3 (first dorsal fin), 4 (pectoral fin), 5 (pelvic fin), 6 (anal fin), 8 (ventrofrontal area), and 12 (eye) were spotted with an average of less than one parasite, while black spots were more abundant for other areas. Values were markedly higher for whiting and pout than for the other species. For example, values higher than 5 were found in the dorsofrontal and dorsoposterior areas (7 and 9) on both sides for whiting and pout, while 1 parasite was observed for other species. Sides do not appear to play a major role for pelagic and demersal species, but this aspect does play a role for flatfishes, which exhibited slightly more metacercariae on the right side, and particularly for dab *L. limanda.* The pelvic fin (area 5) is the only area not affected by metacercariae infection.

### Parasite identification

#### Comparative morphological analysis

**Figure 4:**
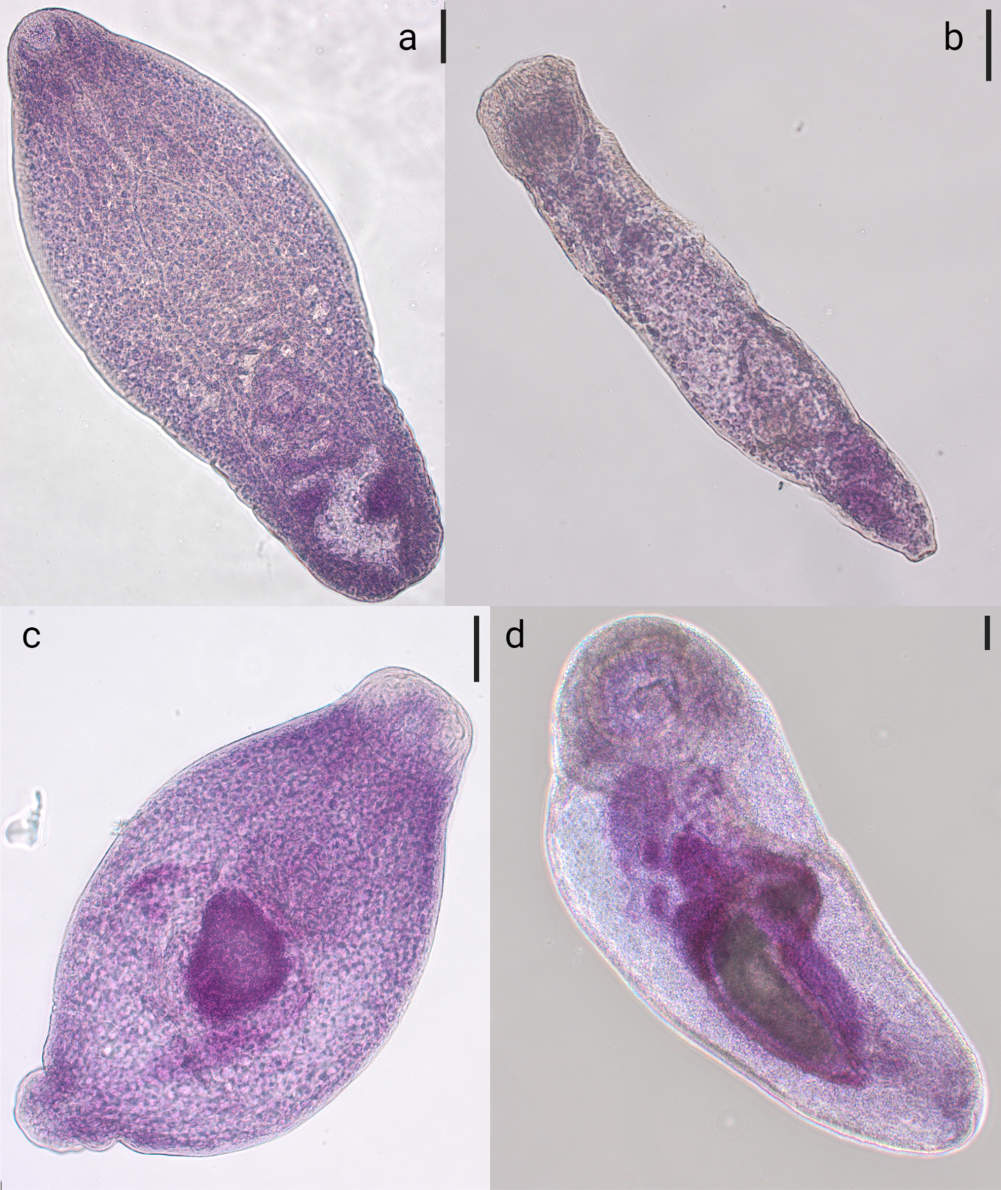
Excysted *Cryptocotyle lingua* metacercariae from whiting of the central North Sea (a) and excysted metacercariae of the family Bucephalidae from pout of the Southern Bight of the North Sea (b) and whiting (c and d) of the eastern English Channel and central North Sea, respectively. Scale: 50 μm.

Four different morphologies were observed (Fig. 4, Table 5) (*n* = 209 parasites). The first one (Fig. 4a) was predominant (*n* = 192 metacercariae) and had the morphological traits of *Cryptocotyle* spp. Excysted metacercariae were linguiform to pyriform, according to their state of contraction at fixation in ethanol, length 0.57 (0.31–0.92) or 0.55 (0.31–0.69) mm for metacercariae from whiting and pout, respectively (Table 4). Width at anterior part of the body was 0.23/0.22 mm (metacercariae from whiting/pout) and second width in the posterior part of the body was 0.14/0.15 mm. The anterior part of metacercariae was covered with scale-like spines, a subterminal oral sucker of 0.06/0.05 mm in length by 0.02/0.02 mm width. The prepharynx was short followed by an elliptical pharynx 0.04/0.04 mm in length by 0.02/0.02 mm in width, with a distance from the oral sucker to the end of pharynx of 0.12/0.11 mm, and distance between the oral sucker and pharynx of 0.02/0.02 mm. Intestinal bifurcation occurred at 0.16/0.16 mm from the oral sucker. The ventral sucker was 0.02/0.02 mm in length and 0.02/ 0.02 mm in width, located on the median line from one-half to two-thirds of the total body length according to contraction. Immature sexual organs were present, in the posterior part of the body.

**Table 4:** Morphometric data of metacercariae with morphological traits of *Cryptocotyle* spp. (*n* = 192) from naturally infected whiting (*n* = 54) and pout (*n* = 26) and bibliographic references. Measurements are expressed in mm (Average (min–max)) and round number at 2 digits.

	Parasites from whiting	Parasites from pout	*Cryptocotyle lingua*	*Cryptocotyle lingua*	*Cryptocotyle lingua*	*Cryptocotyle concava*	*Cryptocotyle jejuna*
Stage of maturity	Metacercariae	Metacercariae	Metacercariae	Adult	Metacercariae	Metacercariae	Metacercariae
Body shape	Linguiform to pyriform	Linguiform to pyriform	/	Linguiform to pyriform	Linguiform to pyriform	Oval	Elongated
Total length	0.57 (0.31–0.92)	0.55 (0.31–0.69)	0.58–0.68	0.55–2.00	0.39–0.92	0.42	/
Width 1	0.23 (0.06–0.32)	0.22 (0.13–0.36)	0.18–0.21	0.20–0.90	0.11–0.31	0.37	/
Width 2	0.14 (0.05–0.39)	0.15 (0.08–0.21)	/	/	0.14–0.25	/	/
Oral sucker length	0.06 (0.03–0.24)	0.05 (0.04–0.08)	0.05–0.06	0.07–0.11	0.03–0.06	0.06	0.05
Oral sucker width	0.06 (0.03–0.28)	0.06 (0.04–0.12)	0.05–0.06	/	/	/	/
Ventrogenital complex length	0.02 (0.01–0.03)	0.02 (0.01–0.05)	0.02–0.03	0.12–0.25	0.02–0.05	/	/
Ventrogenital complex width	0.02 (0.01–0.03)	0.02 (0.01–0.04)	0.02–0.03	/	/	/	/
Distance from oral sucker to end of pharynx	0.12 (0.06–0.16)	0.11 (0.06–0.22)	/	0.03–0.05	0.07–0.12	0.05 (0.01–0.04)	0.03 (±0.01)
Distance between oral sucker and pharynx	0.02 (0.00–0.06)	0.02 (0.00–0.07)	/	/	/	/	/
Intestinal branches	0.16 (0.05–0.31)	0.16 (0.11–0.23)	/	0.28–0.32	0.06–0.19	/	/
Pharynx length	0.04 (0.02–0.06)	0.04 (0.03–0.08)	0.03–0.04	/	/	/	/
Pharynx wide	0.02 (0.01–0.04)	0.02 (0.01–0.05)	0.02–0.03	/	/	/	/
	This study	This study	Borges *et al.* [7]	Ransom [6]	Duflot *et al.* [20]	Goncharov *et al.* [30]	Goncharov *et al.* [30]

The other three morphologies (Figs. 4b–4d) corresponded to a minority of samples (*n* = 17). They exhibited the general traits of family Bucephalidae, such as a translucent, elongated to oval and small- to medium-sized body. An anterior globular attachment organ was present, like an oral sucker or rhynchus, and a ventral sucker absent. The excretory pore was terminal. No further organs could be distinctly observed at this larval stage.

#### Molecular identification

Sequence analysis of the mtDNA *cox1* gene locus (320 bp) was successfully obtained on 1,034 metacercariae out of the 1,909 analyzed, allowing the identification of the parasite species. Likewise, PCR of the *ITS* region of rDNA from 255 parasites (95 identified parasites) resulted in a product of approximately 1,500 bp long. Sequences of the *cox1* gene and *ITS* region were deposited in GenBank under accession numbers MZ731829–MZ731932 and MZ595783–MZ595830, respectively.

BLAST search (Supplementary data 2 and Table 5) of the *cox1* fragments (performed in February to May 2021) led to an average of 99.83% similarity with 4 GenBank sequences corresponding to *C. lingua* samples from Denmark (928 sequences), 99.41% similarity with 13 GenBank sequences corresponding to *C. lingua* from Europe (Ireland, UK, Norway, Denmark, France, and Sweden), Canada and the USA (72 sequences), and 98.67% similarity with a GenBank sequence of *C. lingua* from Russia (14 sequences). Four sequences were identified as *Cryptocotyle concava* with 99.5% similarity with GenBank accession Nos. MT422312 (*C. concava*. Russia: White Sea), and MT422306 (*C. concava*, Varangerfjord, Norway). Four sequences showed only a low similarity value (79%) with sequences of other Heterophyidae (KT883857, *Pholeter gastrophilus* and LC422949, *Metagonimus* sp.). Likewise, three sequences had a low similarity value (74.94%) with AY504855 and AY504859 (*Larval bucephalid* sp.).

**Table 5:** Summary of identifications carried out in this study.

	Morphological method		Molecular method	
	Number of identified parasites	Related percentage of identification	Number of identified parasites	Related percentage of identification
*C. lingua*	192	91.9%	101	98.1%
*C. concava*	/	/	4	0.4%
Bucephalidae	17	8%	3	0.3%
Total	209		1034	

BLAST analysis (Supplementary data 2 and Table 5) of the *ITS* fragments (carried out from April to May 2021) led to an average of 99.8% similarity with 7 GenBank sequences corresponding to *C. lingua* from Danish seas and the English Channel, France (76 sequences). 99.81% similarity was found with 1 GenBank sequence corresponding to *C. lingua* from the White sea. 92.80% similarity was obtained for 1 sequence with a GenBank sequence corresponding to *Bucephalus margaritae* and an average of 88.46% similarity was found for 14 sequences with a GenBank sequence of *Bucephalus polymorphus*. *Cryptocotyle lingua* identifications were confirmed with both markers for 71 individuals.

**Figure 5:**
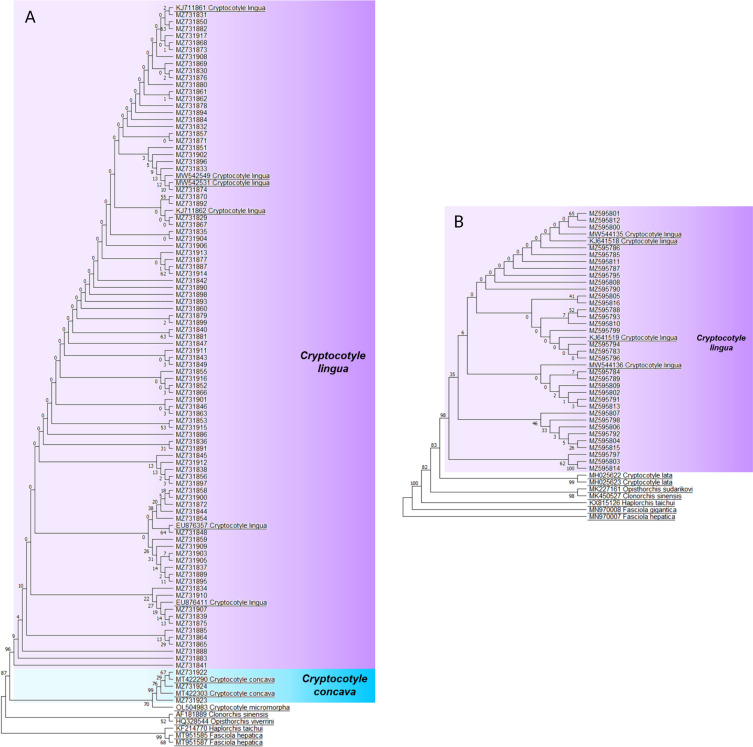
Maximum likelihood trees using 1,000 bootstraps based on *cox1* mtDNA (A) and ITS rDNA (B) sequences of *Cryptocotyle lingua* (purple) and *C. concava* (blue) and reference sequences from GenBank (underlined).

The ML, NJ, ME and Bayesian methods produced phylogenetic trees with similar topologies. Only the ML tree is presented in this manuscript. The *cox1* (Fig. 5A) and *ITS* (Fig. 5B) trees had the highest log likelihoods of −2466.53 and −5763.65 (Fig. 5), respectively. Phylogenetic trees based on nucleotide sequences of the *cox1* gene (Fig. 5A) and *ITS* region (Fig. 5B) showed that all the sequences identified as *C. lingua* by BLAST analysis clustered together in a highly supported clade, including *C. lingua* reference sequences (Fig. 5). The mtDNA *cox1* sequences identified as *C. concava* by BLAST identification also clustered with *C. concava* reference sequences in a monophyletic group (Fig. 5A). Since fewer metacercariae were analyzed for the *ITS* region and *C. concava* was much rarer than *C. lingua* in our sampling, no *ITS* region sequence was retrieved for *C. concava* in the analyzed individuals.

### Geographical distribution of black spot disease and *Cryptocotyle* spp.

**Figure 6:**
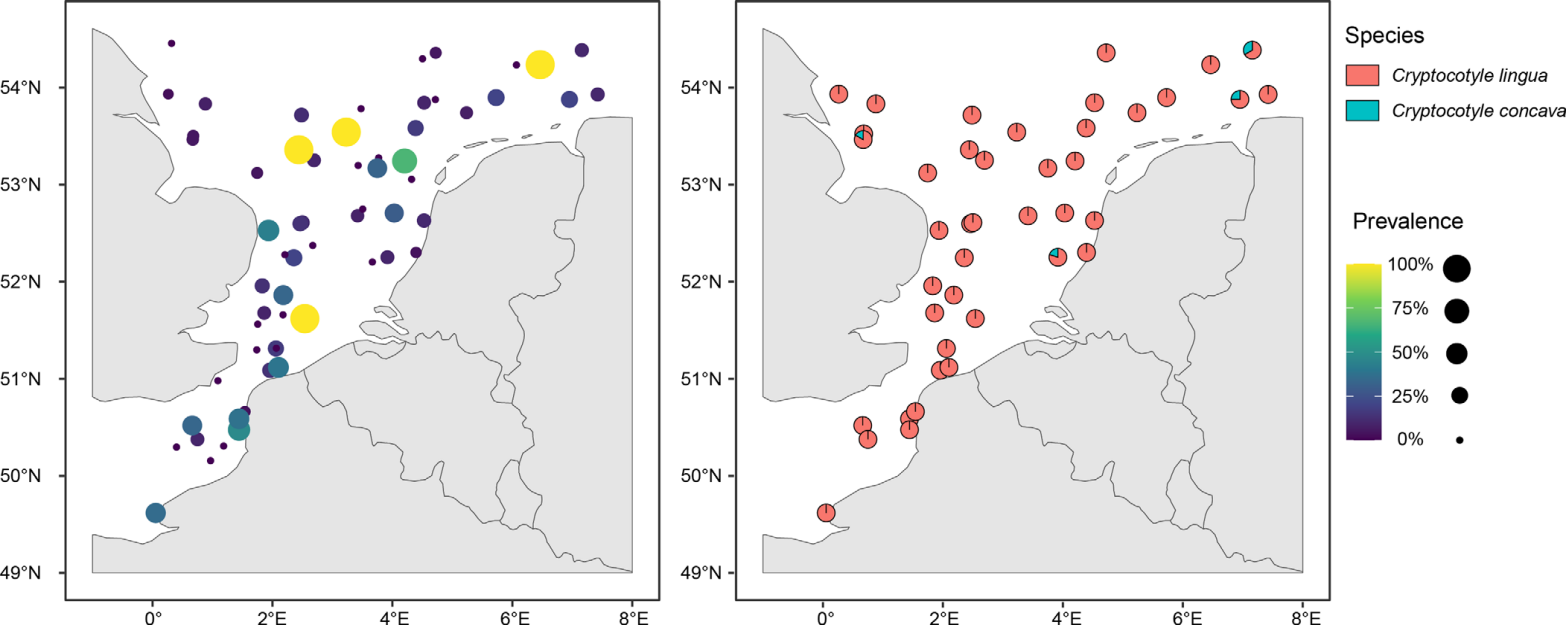
Prevalence of black spot infection (left) and spatial distribution of *Cryptocotyle lingua* and *C. concava* (identified by morphological or molecular methods) (right) by sampling stations in the English Channel and North Sea in January 2019 and 2020. The proportion reflects the sampling size (left) and the percentage of metacercariae identified as *C. lingua* or *C. concava* (right).

Black spot diseases were observed throughout the eastern English Channel and the North Sea (Fig. 6). In the eastern English Channel, 75% of fish with black spots hosted at least one parasite identified as *C. lingua*. This was the most infected geographic area in this study. The Southern Bight of the North Sea was the second geographic area, where 70.45% of parasitized fish hosted at least one parasite identified as *C. lingua*. In the North Sea, rates were lower with 44.32%, 35.23% and 18.18% of fish in the central, eastern and western North Sea, respectively. However, in the eastern and western North Sea, fish parasitized by at least one *C. concava* were found too, with prevalence of 2.27% and 1.14%, respectively (Fig. 6). Only three fish (0.15%) were parasitized with at least one Bucephalidae parasite, and four fish (0.20%) were found to host at least one other Heterophyidae genus than *Cryptocotyle* spp.

### Genetic diversity of *C. lingua* populations

#### Genetic diversity of *C. lingua* populations by geographic area

Estimation of the genetic differentiation of the *C. lingua* specimens by the geographic areas of sampled fish in this study was estimated from the fixation index (*F*_st_) (Table 6). No significant level of differentiation was observed among the overall five geographic areas (*p* < 0.05). However, significant genetic differentiation was found between *C. lingua* from the western North Sea and the eastern English Channel (*F*_st_ = 0.01199; *p* = 0.03306 ± 0.0184*).

**Table 6:** Population pairwise F_st_ from the mtDNA cox1 gene among Cryptocotyle lingua by geographic localities of the eastern English Channel (EEC), Southern Bight of the North Sea (SBNS), eastern North Sea (ENS), central North Sea (CNS), western North Sea (WNS); Below the diagonal: conventional F_st_ from haplotype frequencies; Above the diagonal: p-values of F_st_; significance level 0.05, * = significant value.

	EEC	SBNS	ENS	CNS	WNS
EEC	–	0.09091 ± 0.0262	0.09917 ± 0.0242	0.37190 ± 0.0531	0.03306 ± 0.0184*
SBNS	0.00171	–	0.14050 ± 0.0352	0.85124 ± 0.0432	0.16529 ± 0.0304
ENS	0.00200	0.00241	–	0.38017 ± 0.0544	0.03306 ± 0.0136
CNS	0.00012	−0.00168	0.00027	–	0.23967 ± 0.0361
WNS	0.01199	0.00413	0.00803	0.00310	–

Based on *cox1* (*N* = 998, 272 bp) sequence analyses from the different geographical areas (Table 7), *C. lingua* parasites exhibited 87 haplotypes (Nh). Among the sequences, 80 polymorphic sites (S) were recorded. The sequences of *cox1* from *C. lingua* individuals showed low haplotype diversity (on average *Hd* = 0.476). The same tendency was observed for nucleotide diversity (*π*_cox1_ = 0.00225). The average number of nucleotide differences was also low (*K* = 0.610). The highest haplotype diversity and number of nucleotide differences were observed for *cox1 C. lingua* specimens isolated from the western North Sea (*Hd* = 0.627; *K* = 00793). Similar values of nucleotide diversity were observed in all five defined geographical areas.

**Table 7:** Genetic diversity values of the mtDNA cox1 gene of Cryptocotyle lingua from seven selected fish hosts, by geographic localities of the eastern English Channel (EEC), Southern Bight of the North Sea (SBNS), eastern North Sea (ENS), central North Sea (CNS) and western North Sea (WNS).

	*N*	*Nh*	*π*	*Hd*	*K*	*S*	*Tajima’s D*	*Fu’s Fs*
EEC	298	32	0.00181	0.406	0.493	31	−2,46711***	−56,857
SBNS	278	37	0.00236	0.511	0.643	34	−2,50921***	−63,891
ENS	155	28	0.00268	0.491	0.729	30	−2,52705***	−41,223
CNS	212	27	0.00219	0.474	0.594	25	−2,61775***	−40,894
WNS	55	11	0.00292	0.627	0.793	9	−1,79176 n.s.	−8,171
Total	998	87	0.00225	0.476	0.610	80	−2.61775***	−211.987

Number of analyzed sequences (*N*); number of haplotypes (*Nh*); nucleotide diversity (*π*); haplotype diversity (*Hd*); average number of nucleotide differences (*K*); number of polymorphic sites (*S*); two neutrality tests (Tajima’s D and Fu’s Fs). *p < 0.05; **p < 0.001; ***p < 0.0001; n.s.: not significant.

Tajima’s D and Fu’s Fs values were negative for all fishing geographic areas with significant average: Tajima’s D_*cox1*_ = −2.61775 (*p* < 0.001), Fu’s Fs_*cox1*_ = −211.987. Thus, the null hypothesis of a constant population size (*i.e.*, the population evolves according to the infinite site model and all mutations are selectively neutral) was rejected.

#### Genetic diversity of *C. lingua* populations according to infected fish species

Estimation of the genetic differentiation of the *C. lingua* specimens from different infected fish species was estimated from the fixation index (*F*_st_) (Table 8). No significant level of differentiation was observed among the seven infected fish species (*p* < 0.05).

**Table 8:** Population pairwise F_st_ from mtDNA cox1 gene among Cryptocotyle lingua by fish species from the English Channel and North Sea. Below the diagonal: conventional F_st_ from haplotype frequencies; Above the diagonal: p-values of F_st_; significance level 0.05, * = significant value.

	Herring	Sprat	Whiting	Pout	Dab	Flounder	Plaice
Herring	–	0.44531 ± 0.0174	0.17773 ± 0.0080	0.25977 ± 0.0119	0.47949 ± 0.0149	0.41797 ± 0.0197	0.80078 ± 0.0106
Sprat	0.00011	–	0.09473 ± 0.0103	0.10645 ± 0.0090	0.07129 ± 0.0067	0.07812 ± 0.0094	0.42578 ± 0.0155
Whiting	0.00616	0.03167	–	0.81934 ± 0.0105	0.52148 ± 0.0162	0.08008 ± 0.0081	0.29688 ± 0.0131
Pout	0.00351	0.03053	−0.00101	–	0.41797 ± 0.0152	0.07812 ± 0.0103	0.32129 ± 0.0159
Dab	−0.00081	0.04370	−0.00109	−0.00023	–	0.14941 ± 0.0098	0.45117 ± 0.0126
Flounder	0.00616	0.09117	0.04339	0.04503	0.03482	–	0.19043 ± 0.0097
Plaice	−0.02595	−0.01369	0.01310	0.01114	−0.00376	0.03614	–

Based on *cox1* (*n* = 998, 272 bp) (Table 9) sequence analyses from the different fish species infected by *C. lingua,* parasites exhibited 87 haplotypes (*Nh*). Among the sequences, 80 polymorphic sites (S) were recorded. The sequences of *cox1 C. lingua* specimens showed low haplotype diversity (on average *Hd* = 0.476) with a low nucleotide diversity value (*π*_*cox1*_ *=* 0.00255). The average number of nucleotide differences was low (*K* = 0.610). The highest haplotype diversity and number of nucleotide differences were observed for *cox1 C. lingua* specimens isolated from plaice (*Hd* = 0.786; *K* = 1.000). Similar values of nucleotide diversity were observed in all seven fish species. Few sequences of *C. lingua* were identified from sprat, plaice, and flounder on the *cox1* gene.

**Table 9:** Genetic diversity values of the mtDNA cox1 gene of Cryptocotyle lingua by fish species from the English Channel and North Sea.

	*N*	*Nh*	*π*	*Hd*	*K*	*S*	*Tajima’s D*	*Fu’s Fs*
Herring	31	13	0.00419	0.735	1.140	13	−2,23666**	−11,230
Sprat	12	4	0.00273	0.636	0.742	3	−0,82879 n.s.	−1,256
Whiting	642	61	0.00216	0.4630	0.588	57	−2,58160***	−131,969
Pout	251	28	0.00217	0.474	0.588	27	−2,39500**	−42,541
Dab	44	9	0.00214	0.405	0.582	10	−2,18614**	−7,415
Flounder	10	3	0.00204	0.378	0.556	2	−0,69098 n.s.	−0,594
Plaice	8	5	0.00368	0.786	1.000	4	−1,53470 n.s.	−2,800
Total	998	87	0.00225	0.476	0.610	80	−2.61775***	−211.987

Number of analyzed sequenced (*N*); number of haplotypes (*Nh*); nucleotide diversity (*π*); haplotype diversity (*Hd*); average number of nucleotide differences (*K*); number of polymorphic sites (*S*); two neutrality tests (*Tajima’s D* and *Fu’s Fs*)*;* *p < 0.05; **p < 0.001; ***p < 0.0001; n.s. not significant.

Tajima’s D and Fu’s Fs values were negative for all fishing geographical areas with significant average, Tajima’s D_*cox1*_ = −2.61775 (*P* < 0.001), Fu’s Fs _*cox1*_ = −211.987. Thus, the null hypothesis of a constant population size (*i.e.*, the population evolves according to the infinite site model and all mutations are selectively neutral) was rejected.

### Relationship among haplotypes of *C. lingua*

**Figure 7:**
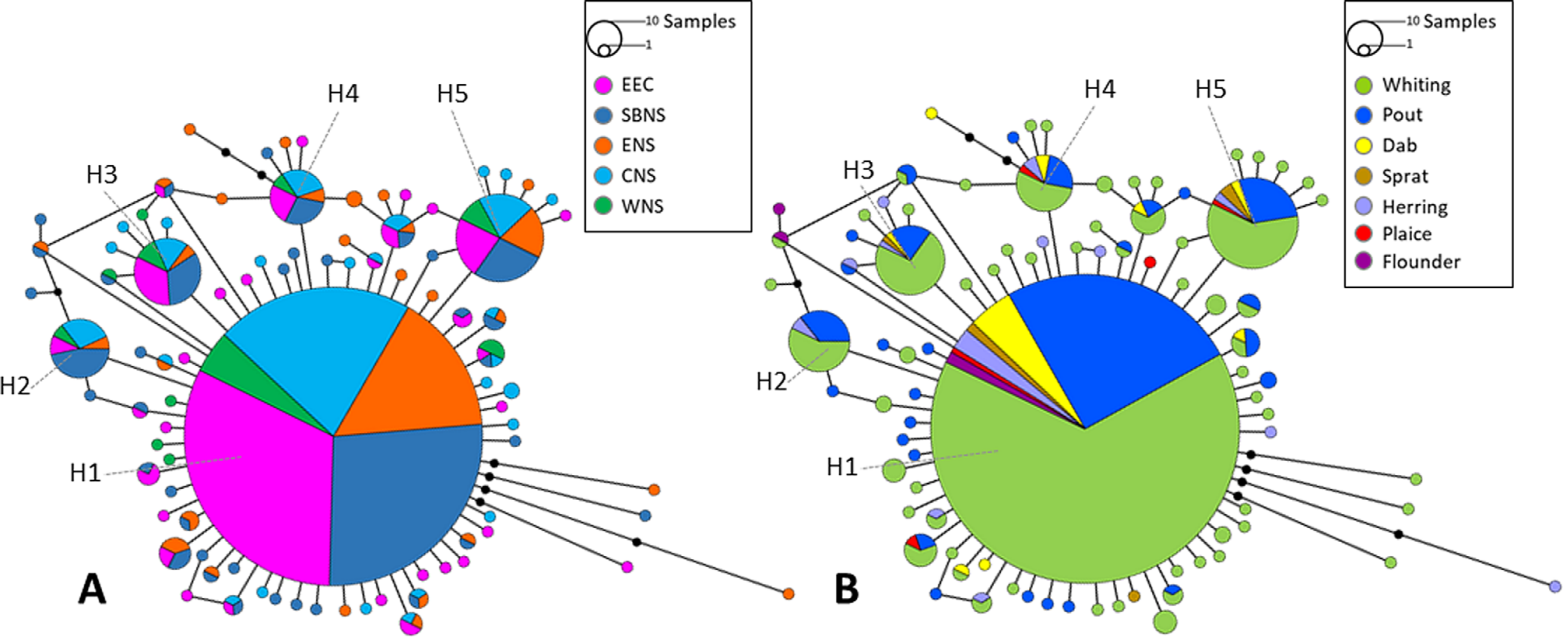
Haplotype networks of *Cryptocotyle lingua* based on cox1 gene sequences (*n* = 998 sequences) from seven fish species from the eastern English Channel (EEC)*,* Southern Bight of the North Sea (SBNS)*,* eastern North Sea (ENS)*,* central North Sea (CNS)*,* western North Sea (WNS) (A) and from fish hosts (B)*.* Circles indicate haplotypes*,* their frequencies correspond to the circle sizes*.* The haplotypes linked by a black line differ by one (no black point) or more (number of black points) substitutions*.* Colors are consistent with the map in Figure 1 *.*

The median joining network constructions of *cox1* (Fig. 7) showed the relationship among the 87 haplotypes observed in the 998 parasites identified as *C. lingua*.

There are five major haplotypes (H1 to H5) for *C. lingua cox1* sequences. The major haplotypes were shared by all geographic areas (Fig. 7A). There was only one haplotype that was shared by all host fish species of *C. lingua,* haplotype H1 (Fig. 7B). Equivalent analyses were conducted on the *ITS* region sequences, but no significant results were found on the few sequences available for this analysis.

## Discussion

This is the first study investigating the diversity, prevalence and intensity of encysted metacercariae causing black spots on fish in the eastern English Channel (EEC) and the North Sea (NS). Up to now, many Heterophyidae have been described as responsible for these lesions [43, 53, 75, 76, 88xs]. In this study, seven commercial fish species from the eastern English Channel and the North Sea were characterized by their parasitic ecology of encysted metacercariae inducing black spots. All the studied species were susceptible to encysted metacercarial infection. Encysted metacercariae were found mainly in the skin layer of the fish, and they were also observed on the flesh close to the dorsal fin (areas 7 and 9). These observations are consistent with previous descriptions, which have noted major concentrations of black spots above the lateral line and in the skin layer [7, 20, 21, 59, 85].

Prevalences of infection were highly variable between the fish species and the geographical areas. Plaice, whiting and pout were the most infected species. Prevalence, intensity and abundance trends were not consistent between both sampling years. Nevertheless, the same trends of prevalence between the five geographical areas were observed for whiting and pout. Likewise, Van Den Broek [85] found divergence of intensity and prevalence on pout and whiting samplings between 1973 and 1975 at Kingsnorth Power Station, USA. As previously observed by some authors [21, 55], the black spot immune lesions can be influenced by certain environmental and inherent fish factors. McQueen *et al.* [59] demonstrated that pigmented cells in plaice appeared 10 days after experimental infection, and Chapman and Hunter [17] observed pigmented cells from 10 to 30 days after infection in the cunner *Tautogolabrus adspersus*. Black spot intensity depends on the location of the metacercariae on the fish [17]. Wood and Matthews [89] found that melanization of metacercarial cysts was most intense in fish maintained in constant darkness after cercarial infection. In the present study, parasitological descriptors were estimated on visible black spot counts. Hence, false negatives were probably included in this numbering since black spots can appear only after several days. Recent infestations could be omitted. Experimental infection challenge tests are needed to determine the time and the biotic/abiotic conditions that could influence black spot development in each fish species. Due to the size of this epidemiological study, based on more than 1,500 fish, it was technically difficult to isolate all the encysted parasites from each individual fish. As this study is designed to describe parasites causing black spot diseases in marine fish and as Duflot *et al.* [21] described the ratio between black spot and recovered metacercariae as close to one, black spot counting was used to estimate parasitical descriptors of this substantial sampling. To strengthen these results, identifications of metacercariae were performed. For flatfish, few metacercariae were identified in comparison to the number of black spots observed. As flatfish possess chromatophores on the dermal layer for differential responsiveness to backgrounds [11], their melanophores are more mobile, so those around metacercariae could migrate after infection. On the other hand, the presence of these chromatophores could be mistaken for black spots due to encysted parasites. This observation could explain the discrepancy between the high intensity and the low number of identified parasites observed on flounder.

A total of 1,586 fish were examined and 325 of them were parasitized by encysted metacercariae. A subsample of the metacercariae from each sample were isolated and identified by morphological traits or molecular tools. A total of 209 excysted metacercariae were successfully identified by the morphological method. Four morphologies were observed according to the morphological characteristics described by several authors [7, 13, 27, 30, 53, 66, 80, 83]. The majority of metacercariae were identified as *C. lingua* after comparison with published general traits and measurements of the main characteristic organs [7, 66]. In fact, observed metacercariae differed from *C. concava* and *C. jejuna* by their body shape and their width. Three other morphologies were observed. Some larvae with morphological characteristics of the Bucephalidae family were observed [27]. Due to the scarcity of detailed identification-keys for the genus and species of this family, parasites were only identified to the family level. Additionally, Bower [8] highlighted the lack of studies on their life cycle and their taxonomy. From the description of Baturo (1977) in Kinkelin *et al.* [42] and Al-Zubaidy [2], some metacercariae (Fig. 4, b) could belong to *Bucephalus polymorphus* or *Bucephalus margaritae*. Other metacercariae (Fig. 4, d) could belong to the *Rhipidocotyle* genus with regards to the description of Gibson [27] and Bartoli and Bray [4]. The last morphology (Fig. 4, c) observed could not be related to any former description. However, these observations were consistent with those of Nicoll [60], who observed Bucephalidae parasites in different fish species from the English Channel on the British coast. The morphological identifications were strengthened and confirmed by molecular identification carried out on different parasites isolated from the same host. Nevertheless, as excysted metacercariae are small parasites (less than 500 μm), it was impossible to undertake morphology and molecular identification on the same individual.

A total of 1,034 and 95 specimens were identified by molecular methods with *cox1* and *ITS* markers, respectively. These two markers were selected because the available sequences targeted mainly the *cox1* and *ITS* region within the superfamily Opisthorchioidea [22]. Some molecular identifications of isolated metacercariae were not successful. Due to COVID-19 lockdowns, some samples were kept frozen for a long time and frost could have damaged the DNA. According to BLAST analysis, three main identifications were obtained with the *cox1* marker: *C. lingua, C. concava* and larval of bucephalid spp. Results of the *ITS* region identification confirmed *cox1* results with identifications of *C. lingua, Bucephalus polymorphus* and *B. margaritae*. *Cryptocotyle lingua* identifications were confirmed by both selected markers for 71 metacercariae. The newly generated sequences of *C. lingua* on *cox1* and *ITS* markers clustered with strong bootstrap values with the reference sequences of *C. lingua*. In both BLAST analysis and phylogenetic trees, the majority of studied sequences with both molecular markers revealed high similarity with both *C. lingua* cercariae and metacercariae sampled in the North of Europe and in North America [6, 7, 20] and some sequences matched with *C. lingua* from *Littorina littorea* isolated in the White Sea (Kartesh, Russia) [31]. The position of the Opisthorchiidae reference sequences (*Clonorchis* and *Opisthorchis* sp.) used in the phylogenies confirms the question of the problematic nature of interrelationships between Opisthorchioidea. The present phylogenies were consistent with the results of Tatonova and Besprozvannykh [83], Kuzmina *et al.* [46] and Sokolov *et al.* [79], which pointed out the controversial phylogenetic positions of the family and genera of the superfamily Opisthorchioidea. Moreover, Kacem *et al.* [41] observed that the Heterophyidae and Opisthorchiidae form an inseparable single clade. The position of the *Cryptocotyle* genus is also controversial, sometimes characterized as Heterophyidae [13, 14, 30] and sometimes as Opisthorchiidae [79, 83]. These data, in addition to those of the present study, confirm the need for additional descriptions using both adult stage morphological identification and molecular confirmation to constitute reliable and comparable data for further studies.

Eventually, the morphological and molecular identifications were in accordance. *Cryptocotyle lingua* was the main species causing black spot diseases identified in the English Channel and the North Sea ecosystems. Thereby, the prevalence of *Cryptocotyle lingua* can be assessed by the prevalences obtained by counting black spot in these marine ecosystems. Some *Cryptocotyle concava* and Bucephalidae larvae were also obtained, showing that encysted metacercariae of other species coexist in these ecosystems. The low infestation levels of these taxa could be due to the selected anatomical sampling. Indeed, the most visible infected area was chosen here and was mostly represented by an area of the flesh (Nos. 7, 8, 9 or 10), but the preferential site of infection for *Cryptocotyle concava* and Bucephalidae parasites was not always the one selected for this study. *Cryptocotyle concava* predominantly infects the kidney, the fins, the gills and the skin of fish hosts [30, 91]. The Bucephalidae have been described as encysting in deeper tissues in fish, such as cranial nerves [87].

*Cryptocotyle lingua* was distributed in all the studied species of the ecosystem of the eastern English Channel and the North Sea. *Cryptocotyle concava* was observed on flounder and plaice near the British and Netherlands coasts. These observations agreed with the detection of *C. concava* on gobies *Pomatoschistus microps* in Stiffkey (Norfolk, UK) by El-Mayas and Kearn [23] or on *P. microps* and *Pomatoschistus minutus* by Malek [57]. *Cryptocotyle concava* is a marine species, but is also observed in brackish, fresh and terrestrial waters [90]. *Cryptocotyle lingua* was observed in *Littorina littorea* in Cardigan Bay [69, 70] or in Cawsands, Plymouth [58]. In addition, *C. lingua* was observed in the North of Europe, at the metacercariae stage in *Chelon labrosus* (Plymouth, United Kingdom) [88], *Gadus morhua* (Danish waters) [7, 20] or *Merlangius merlangus* (English Channel, Bristol Channel and Inner Severn Estuary) [20, 62].

Mixed infections were observed in this study. As previously discussed, the presence of trematodes different from *Cryptocotyle* might have been underestimated due to the sampling strategy. Further analysis on other anatomical areas of fish will make it possible to specify the Bucephalidae and *C. concava* infection levels in the eastern English Channel and North Sea ecosystems. Coinfections of encysted metacercariae could amplify the severity and the dynamics of fish diseases, where each parasite has its own effects [56]. Moreover, parasitic coinfection could lead to competition of parasites against each other for tissue tropism inside the infected host [56].

A total of 998 and 72 specimens of *C. lingua* were investigated for their genetic diversity on *cox1* and *ITS*, respectively. The weak genetic differentiation between the five geographic areas suggests the existence of a low level of genetic differentiation between the *C. lingua* metacercariae of the English Channel and North Sea. Moreover, no specific diversity has been observed within the different intermediate fish hosts of *Cryptocotyle lingua*, which could confirm that these parasites were not specific in the choice of this intermediate host [88]. To our knowledge, only Blakeslee *et al.* [6] and Gonchar [29] have previously studied the genetic diversity of *cox1 Cryptocotyle* spp. cercariae in North America and in the White Sea (Russia), respectively. Both have found minimal differentiation between location sites. Blakeslee *et al.* [6] demonstrated a reduction in genetic diversity in North America *vs.* Europe. It would be interesting to expand such a study on the genetic diversity of *C. lingua* to broader geographical areas with ecosystems significantly different from each other.

To conclude, this study described high prevalence and intensity values of encysted metacercariae infection from both regions: the eastern English Channel and the North Sea. The major taxon observed was *Cryptocotyle* spp. Two species, *C. lingua* and *C. concava*, were identified in these ecosystems. All examined fish species and all studied geographic areas were subject to encysted metacercariae infections.

This broad distribution brings into focus a potential risk from a public health point of view. The *Cryptocotyle* genus has been described as zoonotic [15] and fish-borne zoonotic trematodes are gaining public attention worldwide, with more than 18 million people infected annually [56]. Moreover, so far, the fishing industry is not aware of the presence of this parasite and black spots are not considered a parasitological risk. Thus, although European legislation bans the sale for human consumption of fishery products that are obviously contaminated with parasites, seafood professionals do not discard fish speckled with black spots. Furthermore, new sampling needs to be carried out to assess the potential risk for consumer and human health. Similarly, from an ecological point of view, the abiotic and biotic factors influencing *Cryptocotyle* spp. distribution should be analyzed to have a better understanding of the circulation of these parasites in a marine ecosystem.

## Supplementary materials

The supplementary material of this article is available at https://www.parasite-journal.org/10.1051/parasite/2023028/olm. *Supplementary data 1*: Infection with encysted metacercariae reporting data of the different infected samples, according to fish areas: (1) caudal fin, (2) 2nd dorsal fin, (3) 1st dorsal fin, (4) pectoral fin, (5) pelvic fin, (6) anal fin, (7) dorsofrontal area, (8) ventrofrontal area, (9) dorsoposterior area, (10) ventroposterior area, (11) opercula, and (12) eye. Bold letters indicate cases in which the average numbers of observed black spots are the 5th highest of each fish species (R: right side, L: left side). *Supplementary data 2*: Summary of BLAST analysis results on the *cox1* gene and ITS region of rDNA sequences; *: number of sequences from parasites isolated in the present study. *Supplementary data 3*: Haplotype network groups of *C. lingua* for *cox1* and ITS and their number of sequences.
