# Supplementary material for: Black spot diseases in seven commercial fish species from the English Channel and the North Sea: infestation levels, identification and population genetics of Cryptocotyle spp
Source: Parasite. 2023 Jul 6;30:28. doi: 10.1051/parasite/2023028 (PMC10327545; doi:10.1051/parasite/2023028)
Supplement: Supplementary file 1 — Supplementary data 1: Infection with encysted metacercariae reporting data of the different infected samples, according to fish areas: (1) caudal fin, (2) 2nd dorsal fin, (3) 1st dorsal fin, (4) pectoral fin, (5) pelvic fin, (6) anal fin, (7) dorsofrontal area, (8) ventrofrontal area, (9) dorsoposterior area, (10) ventroposterior area, (11) opercula, and (12) eye. Bold letters indicate cases in which the average numbers of observed black spots are the 5th highest of each fish species (R: right side, L: left side). Supplementary data 2: Summary of BLAST analysis results on the cox1 gene and ITS region of rDNA sequences; *: number of sequences from parasites isolated in the present study. Supplementary data 3: Haplotype network groups of C. lingua for cox1 and ITS and their number of sequences. [file parasite-30-28-s1.pdf]

Supplementary Material

Supplementary data 1: Infection with encysted metacercariae reporting data of the different infected samples, according to fish areas : (1) caudal fin, (2) 2nd dorsal fin, (3) 1st dorsal fin, (4) pectoral fin, (5) pelvic fin, (6) anal fin, (7) dorsofrontal area, (8) ventrofrontal area, (9) dorsoposterior area, (10) ventroposterior area, (11) opercula, and (12) eye. Bold letters indicate cases in which the average numbers of observed black spots are the 5th highest of each fish species (R: right side, L: left side).

	R/L1	R/L2	R/L3	R4	R/L5	R6	R7	R8	R9	R10	R11	R12	L4	L6	L7	L8	L9	L10	L11	L12
Whiting	1.6	0.6	1.3	1.3	0.0	0.2	**6.6**	1.0	**8.5**	2.2	**3.0**	0.4	1.0	0.0	**6.9**	1.1	**8.2**	1.5	**3.0**	0.5
Sprat	**0.1**	**0.1**	0.0	0.0	0.0	0.0	0.0	**0.1**	**0.7**	**0.1**	0.0	0.0	0.0	0.0	0.1	0.0	**0.3**	**0.1**	0.0	0.0
Herring	0.0	0.0	0.0	0.0	0.0	0.0	**0.7**	0.0	**0.3**	0.0	0.0	0.0	0.0	0.0	**0.5**	0.0	**0.3**	0.0	0.0	0.0
Pout	1.0	0.9	1.5	1.0	0.1	0.4	**5.5**	1.0	**5.8**	1.6	**1.8**	0.2	0.8	0.0	**5.0**	1.3	**5.5**	1.6	1.5	0.2
Dab	**0.2**	**0.2**	0.0	0.1	0.0	0.1	**0.2**	0.1	**1.4**	**1.6**	0.1	0.0	0.0	0.0	0.0	0.0	0.1	0.1	0.0	0.0
Flounder	**2.9**	**1.0**	0.0	0.1	0.1	**0.9**	0.2	0.0	**1.2**	**1.2**	0.0	0.0	0.1	0.0	0.2	0.1	0.4	0.5	0.1	0.0
Plaice	**0.8**	**0.3**	0.0	**0.1**	0.0	**0.2**	**0.1**	0.0	0.0	0.0	**0.4**	0.0	0.0	0.0	0.0	0.0	**0.1**	0.0	0.0	**0.1**
All species	1.4	0.6	0.8	0.7	0.0	0.4	**3.7**	0.6	**4.8**	**1.5**	**1.5**	0.2	0.6	0.0	**3.7**	0.7	**4.4**	1.0	**1.5**	0.2

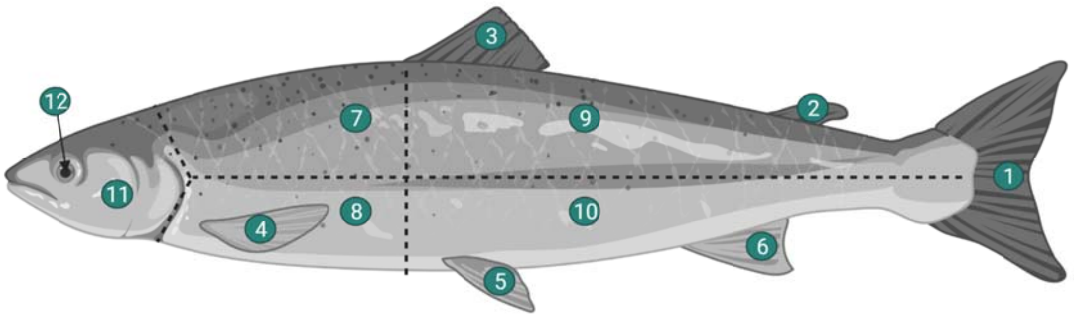

Supplementary data 2: Summary of BLAST analysis results on the *cox1* gene and ITS region of rDNA sequences; *: number of sequences from parasites isolated in the present study.

	Accession numbers	Reference	Average Query cover	Average % identity	Nb of sequences*
***cox1* gene**					
C. lingua	EU876357, EU876365, EU876374, EU876382, EU876409, EU876411, EU876423, EU876427, EU876435, EU876457, EU876489, EU876496, EU876497, EU876502	Blakeslee *et al.* [1]	98%	99.41%	72
	KJ711862, KJ711864, KJ711865, KJ711866	Borges *et al.* [2]	99%	99.83%	928
	MW361241	Gorbushin and Tolstenkov [6]	99%	98.67%	14
C. concava	MT422312, MT422306	Gonchar [5]	86%	99.50%	4
Langeronia macrocirra	HM853563	Martínez-Salazar and León-Règagnon [9]	41.9%	80.22%	6
Neoplagioporus elongatus	LC196193.1	Yano and Urabe [14]	54%	80.21%	1
Pholeter gastrophilus	KT883857	Fraija-Fernández *et al.* [4]	65.98%	79.88%	3
Larval bucephalid spp.	AY504855, AY504859	Hutson *et al.* [7]	92.67%	74.94%	3
Dicrocoeliidae spp.	MG845911	Waki *et al.* [12]	88%	76.07%	2
Metagonimus sp.	LC422949	Nakao (Unpublished)	95%	79.07%	1
***ITS* region of rDNA**					
C. lingua	MW544136, MW544128, MW544135, MW544118, MW544112, MW544139,	Duflot *et al.* [3]	100%	99.8%	76
	MW361240	Gorbushin and Tolstenkov [6]	98%	99.81%	1
Bucephalus margaritae	KT273395	Nolan *et al.* [10]	99%	92.80%	1
Bucephalus polymorphus	AY289238	Stunženas *et al.* [11]	99%	88.46%	14
Prosorhynchoides megacirrus	KT273391	Nolan *et al.* [10]	100%	93.99%	1
Timoniella spp.	MF491832	Kvach *et al.* [8]	56%	99.87%	1
Metorchis orientalis	MT231323	Wang *et al.* [13]	95%	82.24%	1

Supplementary data 3: Haplotype network groups of *C. lingua* for *cox1* and ITS and their number of sequences.

**Name of the haplotype group**	**Number of sequences in the group**	**Name of the haplotype group**	**Number of sequences in the group**
COX_01	3	COX_45	1
COX_02	1	COX_46	1
COX_03	718	COX_47	1
COX_04	3	COX_48	1
COX_05	1	COX_49	3
COX_06	2	COX_50	1
COX_07	1	COX_51	1
COX_08	28	COX_52	1
COX_09	62	COX_53	3
COX_10	24	COX_54	1
COX_11	36	COX_55	1
COX_12	1	COX_56	2
COX_13	1	COX_57	1
COX_14	1	COX_58	1
COX_15	2	COX_59	1
COX_16	9	COX_60	1
COX_17	1	COX_61	1
COX_18	6	COX_62	1
COX_19	1	COX_63	1
COX_20	8	COX_64	1
COX_21	4	COX_65	1
COX_22	2	COX_66	1
COX_23	1	COX_67	1
COX_24	4	COX_68	1
COX_25	2	COX_69	1
COX_26	2	COX_70	1
COX_27	3	COX_71	1
COX_28	1	COX_72	1
COX_29	4	COX_73	1
COX_30	1	COX_74	1
COX_31	1	COX_75	1
COX_32	1	COX_76	1
COX_33	2	COX_77	1
COX_34	1	COX_78	1
COX_35	1	COX_79	1
COX_36	2	COX_80	1
COX_37	1	COX_81	1
COX_38	1	COX_82	1
COX_39	1	COX_83	1
COX_40	1	COX_84	1
COX_41	1	COX_85	1
COX_42	1	COX_86	1
COX_43	1	COX_87	1
COX_44	2		

References

[1] Blakeslee AMH, Byers JE, Lesser MP. 2008. Solving cryptogenic histories using host and parasite molecular genetics: the resolution of *Littorina littorea*'s North American origin. Molecular Ecology, 17(16), 3684–3696.

[2] Borges JN, Skov J, Bahlool QZM, Møller OS, Kania PW, Santos CP, Buchmann K. 2015. Viability of *Cryptocotyle lingua* metacercariae from Atlantic cod (*Gadus morhua*) after exposure to freezing and heating in the temperature range from −80 °C to 100 °C. Food Control, 50, 371–377.

[3] Duflot M, Gay M, Midelet G, Kania PW, Buchmann K. 2021. Morphological and molecular identification of *Cryptocotyle lingua* metacercariae isolated from Atlantic cod (*Gadus morhua*) from Danish seas and whiting (*Merlangius merlangus*) from the English Channel. Parasitology Research, 3417–3427.

[4] Fraija-Fernández N, Fernández M, Raga JA, Aznar FJ. 2015. Life-history trade-offs in a generalist digenean from cetaceans: the role of host specificity and environmental factors. Parasites & Vectors, 8(1), 659

[5] Gonchar A. 2020. Genetic diversity in monoxenous and trixenous digeneans sharing one molluscan host species. Parazitologiya, 54(6), 491–503.

[6] Gorbushin A, Tolstenkov O. 2020. *Cryptocotyle lingua* curated transcriptome assembly (v1.2.2). Genbank: figshare.

[7] Hutson KS, Styan CA, Beveridge I, Keough MJ, Zhu X, Abs El-Osta YG, Gasser RB. 2004. Elucidating the ecology of bucephalid parasites using a mutation scanning approach. Molecular and Cellular Probes, 18(2), 139–146.

[8] Kvach Y, Bryjová A, Sasal P, Winkler HM. 2018. The taxonomic and phylogenetic status of digeneans from the genus *Timoniella* (Digenea: Cryptogonimidae) in the Black and Baltic seas. Journal of Helminthology, 92(5), 596–603.

[9] Martínez-Salazar EA, León-Règagnon V. 2010. Molecular evidence that *Langeronia macrocirra* and *Langeronia cf. parva* (Trematoda: Pleurogenidae) parasites of anurans from Mexico are conspecific. Mitochondrial DNA, 21(sup1), 3–11.

[10] Nolan MJ, Curran SS, Miller TL, Cutmore SC, Cantacessi C, Cribb TH. 2015. *Dollfustrema durum* n. sp. and *Heterobucephalopsis perardua* n. sp. (Digenea: Bucephalidae) from the giant moray eel, *Gymnothorax javanicus* (Bleeker) (Anguilliformes: Muraenidae), and proposal of the Heterobucephalopsinae n. subfam. Parasitology International, 64(6), 559–570.

[11] Stunženas V, Cryan JR, Molloy DP. 2004. Comparison of rDNA sequences from colchicine treated and untreated sporocysts of *Phyllodistomum folium* and *Bucephalus polymorphus* (Digenea). Parasitology International, 53(3), 223–228.

[12] Waki T, Nakao M, Hayashi K, Ikezawa H, Tsutumi N. 2018. Molecular and morphological discrimination of dicrocoeliid larvae (Trematoda: Digenea) from terrestrial mollusks in Japan. Journal of Parasitology, 104(6), 660–670.

[13] Wang Y, Li X, Sun Q, Gong P, Zhang N, Zhang X, Wang X, Li G, Li J. 2020. First case report of *Metorchis orientalis* from Black Swan. International Journal for Parasitology: Parasites and Wildlife, 13, 7–12.

[14] Yano A, Urabe M. 2017. Larval stages of *Neoplagioporus elongatus* (Goto and Ozaki, 1930) (Opecoelidae: Plagioporinae), with notes on potential second intermediate hosts. Parasitology International, 66(2), 181–185.
